# *MicroRNA-124* negatively regulates *STAT3* to alleviate hypoxic-ischemic brain damage by inhibiting oxidative stress

**DOI:** 10.18632/aging.205513

**Published:** 2024-02-05

**Authors:** Jiaqing Geng, Jiangpeng Feng, Fangzi Ke, Fang Fang, Xiaoqi Jing, Jiaxin Tang, Chengzhi Fang, Binghong Zhang

**Affiliations:** 1 Departments of Neonatology, Renmin Hospital of Wuhan University, Wuhan 430062, China; 2 Central Laboratory, Renmin Hospital of Wuhan University, Wuhan 430062, China; 3 State Key Laboratory of Virology, Modern Virology Research Center, College of Life Sciences, Wuhan University, Wuhan 430062, China

**Keywords:** hypoxic-ischemic brain damage, miR-124, STAT3, oxidative stress, apoptosis

## Abstract

*MicroRNA-124 (miR-124)* is implicated in various neurological diseases; however, its significance in hypoxic-ischaemic brain damage (HIBD) remains unclear. This study aimed to elucidate the underlying pathophysiological mechanisms of *miR-124* in HIBD. In our study performed on oxygen-glucose deprivation followed by reperfusion (OGD)/R-induced primary cortical neurons, a substantial reduction in *miR-124* was observed. Furthermore, the upregulation of *miR-124* significantly mitigated oxidative stress, apoptosis, and mitochondrial impairment. We demonstrated that *miR-124* interacts with the signal transducer and activator of transcription 3 (STAT3) to exert its biological function using the dual-luciferase reporter gene assay. As the duration of OGD increased, *miR-124* exhibited a negative correlation with *STAT3*. *STAT3* overexpression notably attenuated the protective effects of *miR-124* mimics, while knockdown of *STAT3* reversed the adverse effects of the *miR-124* inhibitor. Subsequently, we conducted an HIBD model in rats. *In vivo* experiments, *miR-124* overexpression attenuated cerebral infarction volume, cerebral edema, apoptosis, oxidative stress, and improved neurological function recovery in HIBD rats. In summary, the neuroprotective effects of the *miR-124/STAT3* axis were confirmed in the HIBD model. *MiR-124* may serve as a potential biomarker with significant therapeutic implications for HIBD.

## INTRODUCTION

Neonatal hypoxic–ischaemic encephalopathy (HIE) is a clinical syndrome triggered by various factors, resulting in hypoxia and altered cerebral blood flow [1]. Following the hypoxic–ischaemic event occurring at birth, the initial energy failure phase ensues. As clinical symptoms transiently improve, the brain injury recovery period, also known as the therapeutic window before the secondary energy failure phase, begins [2]. The pathophysiology of neonatal hypoxic–ischaemic brain damage (HIBD) is dynamic, involving key components such as apoptosis, inflammation, excitotoxicity, oxidative stress, and mitochondrial impairment. Due to the disparities between the newborn and adult brain, the neonatal brain is highly sensitive to hypoxia and susceptible to oxidative stress damage due to its elevated oxygen consumption rate, high unsaturated fatty acid content, and low levels of antioxidants and redox-active iron [3]. Therapeutic hypothermia (TH) stands as the only proven treatment for HIE [4]. Several trials have indicated that TH can enhance survival and diminish disability in neonatal HIE. Nevertheless, TH does not prevent adverse outcomes in all neonatal patients. Statistics revealed that the rate of death or disabilities, such as cerebral palsy, epilepsy, developmental delays, and cognitive impairment, is ~50% in hypothermic neonates [5, 6]. Drug therapy and interventions for HIE aim to mitigate the side effects of TH, encompassing the use of morphine and anti-epileptic drugs, correction of neonatal acidosis, appropriate oxygen concentration, monitoring blood glucose levels, reducing jaundice, and minimizing unnecessary interventions [7]. Currently, no drugs have proven effective for HIE, underscoring the imperative to identify and develop effective treatments [8].

Plants, animals, and certain viruses contain a category of small, non-coding single-stranded RNAs known as microRNAs (miRNAs). The discovery of *let-7*, the first miRNA in the early 20^th^ century, marked the beginning of our understanding of these molecules. To date, the miRBase miRNA database (released on 22 March 2018) reports almost 38,589 hairpin pre-miRNAs expressing 48,885 mature miRNAs in 271 species [9]. Notably, ~70% of all miRNAs in the known database are associated with the brain. In the mouse brain, *miR-124* constitutes 25%–48% of total miRNAs, as reported by Lagos-Quintana [10]. *MiR-124* plays a crucial role in controlling the physiological activities of human brain tissue biopsies [11]. Substantial evidence has suggested its importance in physiological processes within the nervous system. The *miR-124*/*SCP1* axis is vital for the neuronal differentiation of adipose-derived stem cells, and *miR-124* stimulates the neuronal differentiation of hair follicle stem cells [12, 13]. Additionally, Xue verified that *miR-9* and *miR-124* inhibited the expression of *Rap2a* transcripts, thereby promoting neuronal dendritic branching and differentiation [14]. Furthermore, *miR-124* regulates cyclic AMP-responsive element-binding protein (CREB) and CREB-mediated signaling during plasticity, influencing long-term plasticity [15]. In neuro-inflammation, *miR-124* is significant, and its overexpression inhibits microglial toll-like receptor 4, thereby regulating microglial activation to reduce neuroinflammation [16]. Reports suggest that *miR-124* can also regulate the heart, kidney, and other organs during ischaemia–reperfusion injury [17, 18].

Based on these previous studies, we designed this experiment to elucidate the role of *miR-124* in neonatal HIBD. We validated the potential mechanisms of *miR-124* in HIBD both *in vitro* and *in vivo*. We expect that these findings will provide valuable insights for formulating treatment strategies for HIBD.

## RESULTS

### Expression of *miR-124* and cell viability in oxygen-glucose deprivation followed by reperfusion (OGD/R)-induced neurons

**Figure 1:**
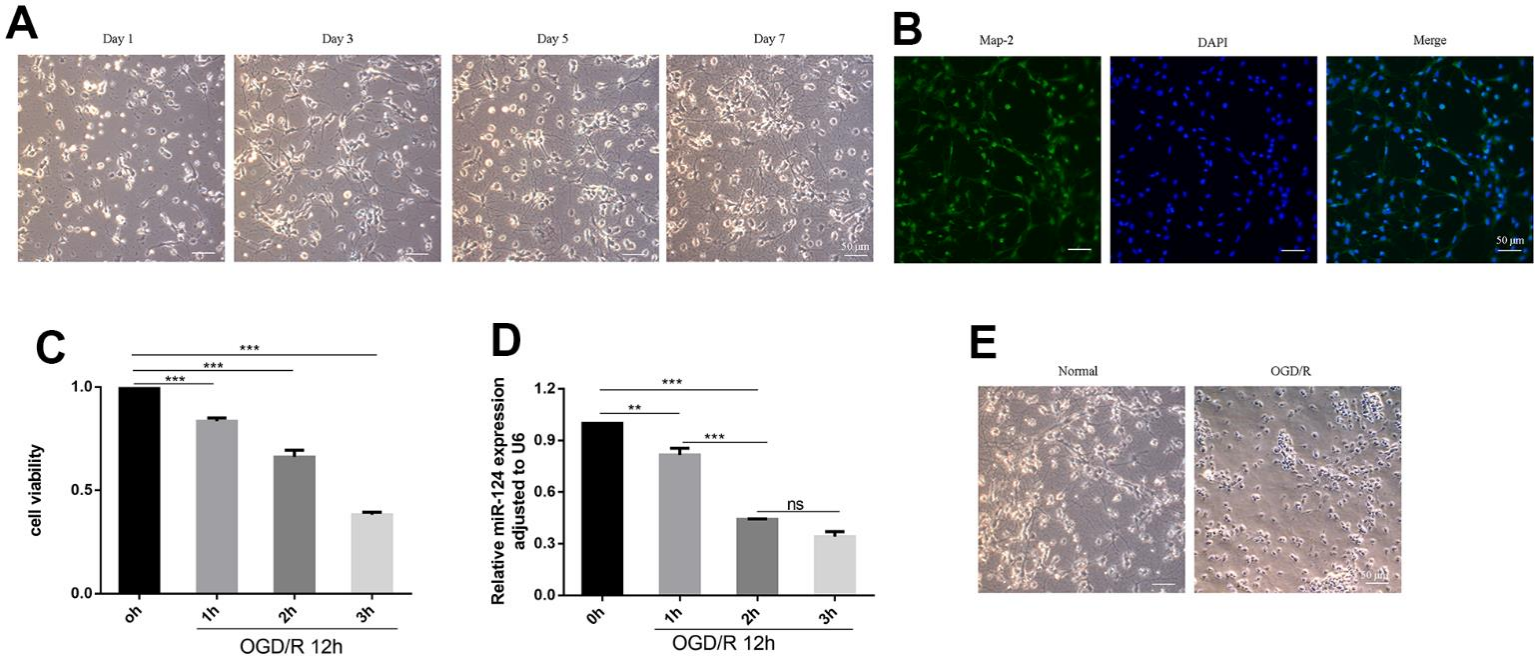
**Expression of *miR-124* and cell viability in OGD/R-induced neurons.** (**A**) The change of primary neurons cultured up to day 1, day 3, day 5, and day 7; (**B**) Representative immunofluorescence images of Map-2 (green) and DAPI (blue) co-staining the primary cortical neurons. Scale bar, 50μm; (**C**) The Cell viability of the primary cortical neurons under OGD treatment for 0, 1, 2, and 3 h were detected by CCK-8 assay; (**D**) qRT-PCR analysis of *miR-124* levels the primary cortical neurons under OGD treatment for 0, 1, 2, and 3 h; (**E**) the change between normal neurons and neurons after OGD(2h)/R treatment. Data were expressed as mean ± SD (derived from three independent experiments for each sample). NS, not significant for *p* > 0.05, * *p* < 0.05, ** *p* < 0.01, and *** *p* < 0.001 (one-way analysis of variance with Tukey’s post hoc tests).

We established an *in vitro* model of HIBD by constructing an OGD/R model using primary cortical neurons. In the experiment, primary neurons cultured up to day 7 which held together via protrusions (Figure 1A). Neurons with a purity exceeding 90% were identified through Map-2 immunofluorescence staining and were utilized for subsequent investigations (Figure 1B). The cell viability of primary cortical neurons was detected by CCK8 assay in different time of OGD. As depicted in Figure 1C, 1D, the cell viability and the *miR-124* levels steadily decreased with an increase in OGD duration (*p* < 0.05). Based on these findings, we hypothesized a connection between *miR-124* and ischemia–hypoxia–reperfusion injury, leading us to select OGD 2 h for subsequent *in vitro* experiments. The changes observed in some neurons after OGD/R treatment (Figure 1E), included blurred cell edges, nuclear fixation, shortened and tortuous protrusions, as well as the disappearance of protrusions.

### The changes of oxidative stress, mitochondrial impairment, and apoptosis in primary neurons with *miR-124* treatment in OGD/R

**Figure 2:**
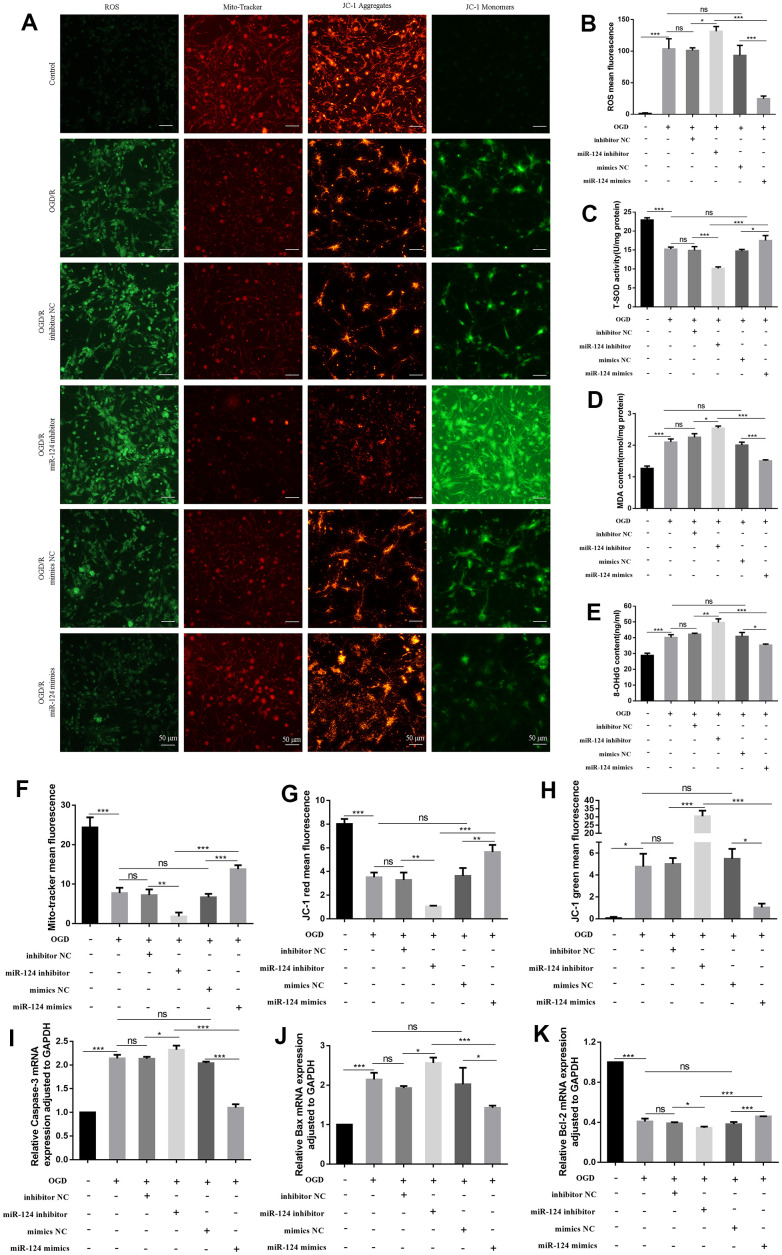
**The changes of oxidative stress, mitochondrial impairment, and apoptosis in primary neurons with *miR-124* treatment in OGD/R.** The neurons were transfected with inhibitor NC, *miR-124* inhibitor, mimics NC, or *miR-124* mimics before OGD/R treatment. (**A**) Intracellular ROS was measured using DCFH-DA with a fluorescence microscope; Representative photomicrograph of mito-tracker red; Representative photomicrographs of fluorescence shift from red to green of JC-1 staining. Scale bar, 50μm; (**B**) Quantitative analysis green fluorescence of ROS; (**C**) The SOD activity was determined by the commercial kits; (**D**) The content of MDA was determined by the commercial kits; (**E**) The content of 8-OHdG was determined by the commercial kits; (**F**) Quantitative analysis of mito-tracker red; (**G**, **H**) Quantitative analysis of fluorescence shift from red to green of JC-1 staining; (**I**–**K**) The mRNA levels of *Caspase-3*, *Bax*, and *Bcl-2* were detected by qRT-PCR. Data were expressed as mean ± SD (derived from three independent experiments for each sample). NS, not significant for *p* > 0.05, * *p* < 0.05, ** *p* < 0.01, and *** *p* < 0.001 (one-way analysis of variance with Tukey’s post hoc tests).

To assess the effect of *miR-124* treatment in OGD/R, we transfected mimics negative control (NC) or *miR-124* mimics, as well as inhibitor NC or *miR-124* inhibitor, prior to OGD/R treatment. As illustrated in Figure 2A, 2B, the ROS DCF fluorescence intensity was significantly higher in OGD/R-treated neurons compared to the normal group, and the *miR-124* inhibitor group exhibited increased DCF fluorescence intensity compared to the NC inhibitor group. Conversely, *miR-124* overexpression attenuated the ROS increase induced by OGD/R treatment compared to the mimics NC group (*p* < 0.05). We also measured superoxide dismutase (SOD) activity, MDA levels, and 8-OHdG levels as sensitive indicators of oxidative damage. OGD/R treatment inhibited SOD activity, while increasing the levels of 8-OHdG and MDA. The *miR-124* inhibitor further inhibited SOD activity and promoted 8-OHdG and MDA content compared to the inhibitor NC group. Conversely, the *miR-124* mimics group exhibited increased SOD activity and decreased 8-OHdG and MDA content compared to the mimics NC group (*p* < 0.05, Figure 2C–2E). In summary, *miR-124* restrained oxidative stress in neurons subjected to OGD/R.

Given that mitochondria are the primary site of oxidative stress in cells, we next assessed mitochondrial impairment [19]. In OGD/R-induced cells, the mito-tracker red probe marked live neurons less efficiently than in the normal group. The mean fluorescence of mito-tracker significantly decreased in the *miR-124* inhibitor group compared to the inhibitor NC group. Conversely, the fluorescence markedly increased in the *miR-124* mimics group compared to the mimics NC group (*p* < 0.05, Figure 2A, 2F). As shown in Figure 2A, 2G, 2H, the red fluorescence changed to green fluorescence in OGD/R-induced cells, suggesting low mitochondrial membrane potential (MMP) compared to the normal group. Additionally, in comparison with the inhibitor NC group, the *miR-124* inhibitor group exhibited an increase in green fluorescence and a decrease in MMP. Conversely, *miR-124* overexpression significantly increased red fluorescence and decreased green fluorescence, stabilizing the potential of the mitochondrial membrane in primary neurons (*p* < 0.05). Our results suggest that *miR-124* overexpression could maintain mitochondrial function.

Oxidative stress is a potent initiator of apoptosis and a key factor in neuronal death. From Figure 2I–2K, the mRNA levels of *Caspase-3* and *Bax* notably increased in the OGD group compared to the normal group. The *miR-124* inhibitor increased the levels of *Caspase-3* and *Bax* mRNA compared to the control group, while the *miR-124* mimics group exhibited reduced *Caspase-3* and *Bax* mRNA levels compared to the mimics NC group. However, the *Bcl-2* mRNA levels were significantly increased in the *miR-124* mimics group compared to the mimics NC group, revealing a negative relationship between *Bcl-2* mRNA levels and *Bax* mRNA levels (*p* < 0.05). In summary, *miR-124* overexpression can alleviate oxidative stress, mitochondrial impairment, and apoptosis in neurons following OGD/R treatment.

### *STAT3* identified as a direct target gene of *miR-124*

**Figure 3:**
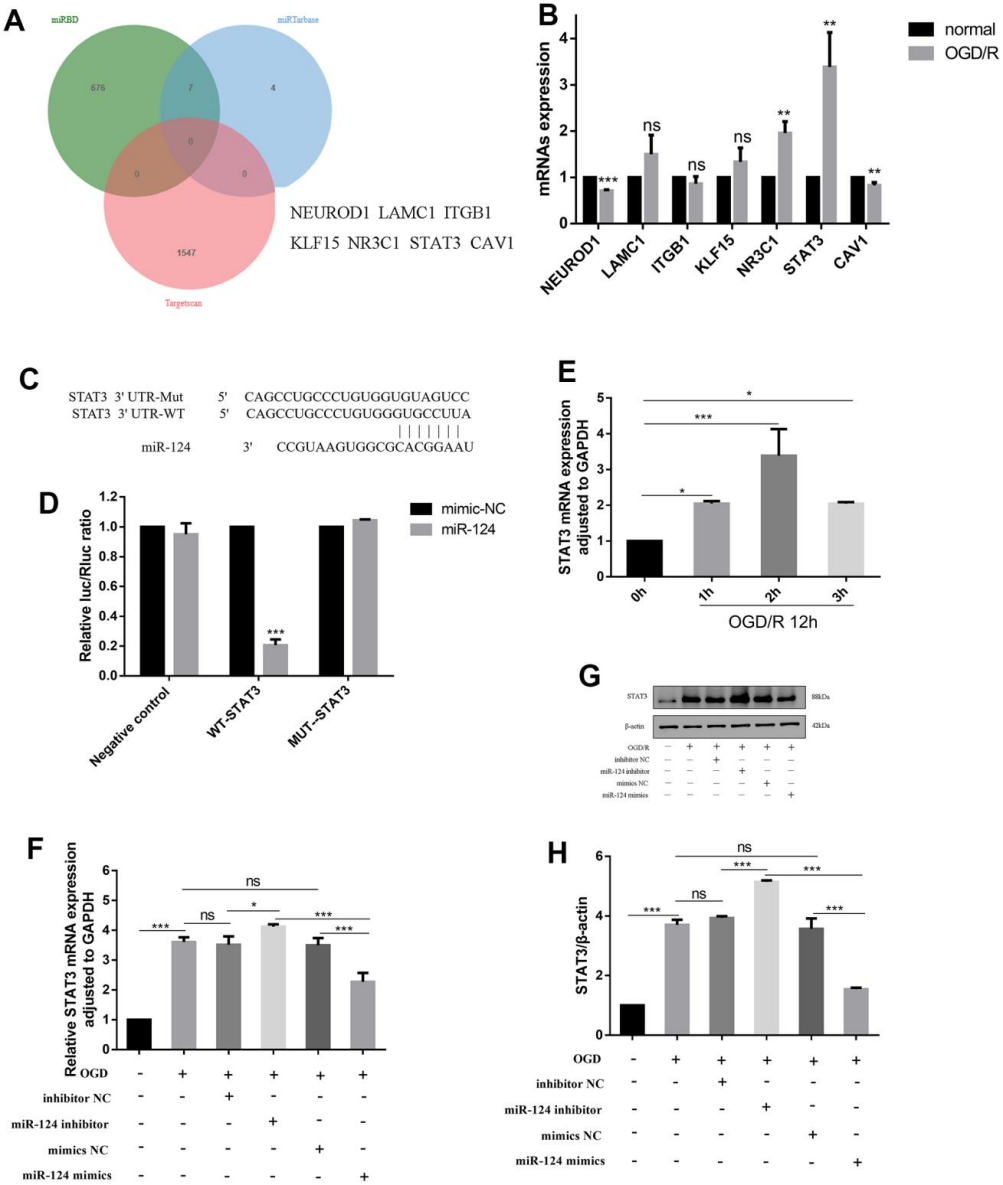
***STAT3* identified as a direct target gene of *miR-124*.** (**A**) miRTarBase, miRDB and TargetScan were used to predict the potential targets of *miR-124* and found the potential target genes (*NEUROD1, LAMC1, ITGB1, KLF15, NR3C1, STAT3, CAV1*); (**B**) The mRNAs expression levels of *miR-124* potential targets; (**C**, **D**) The predicted binding sites between *miR-124* and *STAT3* according to the TargetScan; The neurons were randomly divided into 6 groups, i.e., vector+ mimics NC, vector +*miR-124* mimics, *STAT3*-WT + mimics NC, *STAT3*-WT + *miR-124* mimics, *STAT3*-Mut + mimics NC, and *STAT3*-Mut + *miR-124* mimics. The relative luciferase activity was measured using a dual-luciferase reporter system; (**E**, **F**) The *STAT3* mRNA was detected by qRT-PCR; (**G**) The protein levels of STAT3 was detected by Western blot; (**H**) Quantitative analysis of the protein levels of STAT3; Data were expressed as mean ± SD (derived from three independent experiments for each sample). NS, not significant for *p* > 0.05, * *p* < 0.05, ** *p* < 0.01, and *** *p* < 0.001 (Student’s t-test (**B**, **D**), or one-way analysis of variance with Tukey’s post hoc test (**E**, **F**, **H**)).

To identify potential targets of *miR-124*, we utilized three databases (miRTarBase, miRDB, and TargetScan) and identified *NEUROD1*, *LAMC1*, *ITGB1*, *KLF15*, *NR3C1*, *STAT3*, and *CAV1* as potential target genes of *miR-124* (Figure 3A). Subsequently, we examined the expression of these potential targets by qRT-PCR and found that, after OGD/R treatment, *NEUROD1* mRNA decreased, while *NR3C1*, *STAT3*, and *CAV1* mRNA were up-regulated (*p* < 0.05, Figure 3B). Notably, the level of *STAT3* mRNA changed significantly in OGD/R-induced cells. Consequently, we selected *STAT3* as the target gene of *miR-124* for further investigation, finding that it could be targeted by *miR-124* in the 3′-untranslated region (UTR) according to TargetScan (Figure 3C). Moreover, the relationship between *STAT3* and *miR-124* was validated using a dual-luciferase reporter assay. The results showed a significant reduction in luciferase activity when *miR-124* mimics were co-transfected with 3′-UTR-WT of the *STAT3* reporter, confirming that *miR-124* inhibits *STAT3* expression by binding to its mRNA 3′-UTR (Figure 3D). In Figure 3E, *STAT3* mRNA gradually increased with the extension of hypoxia duration, reaching its peak at OGD 2 h, indicating a negative correlation between *miR-124* and *STAT3* changes (*p* < 0.05). Furthermore, the *STAT3* mRNA and protein levels were significantly elevated in OGD/R-treated neurons compared to the normal group, as revealed by qRT-PCR and western blotting. Correspondingly, in the *miR-124* inhibitor group, *STAT3* mRNA and protein levels were higher than in the inhibitor NC group. Conversely, *miR-124* mimics reversed these effects (*p* < 0.05, Figure 3F–3H). In summary, *miR-124* may exert its biological function by targeting *STAT3* in OGD/R-induced neurons.

### *STAT3* overexpression promotes oxidative stress in OGD/R-treated neurons

**Figure 4:**
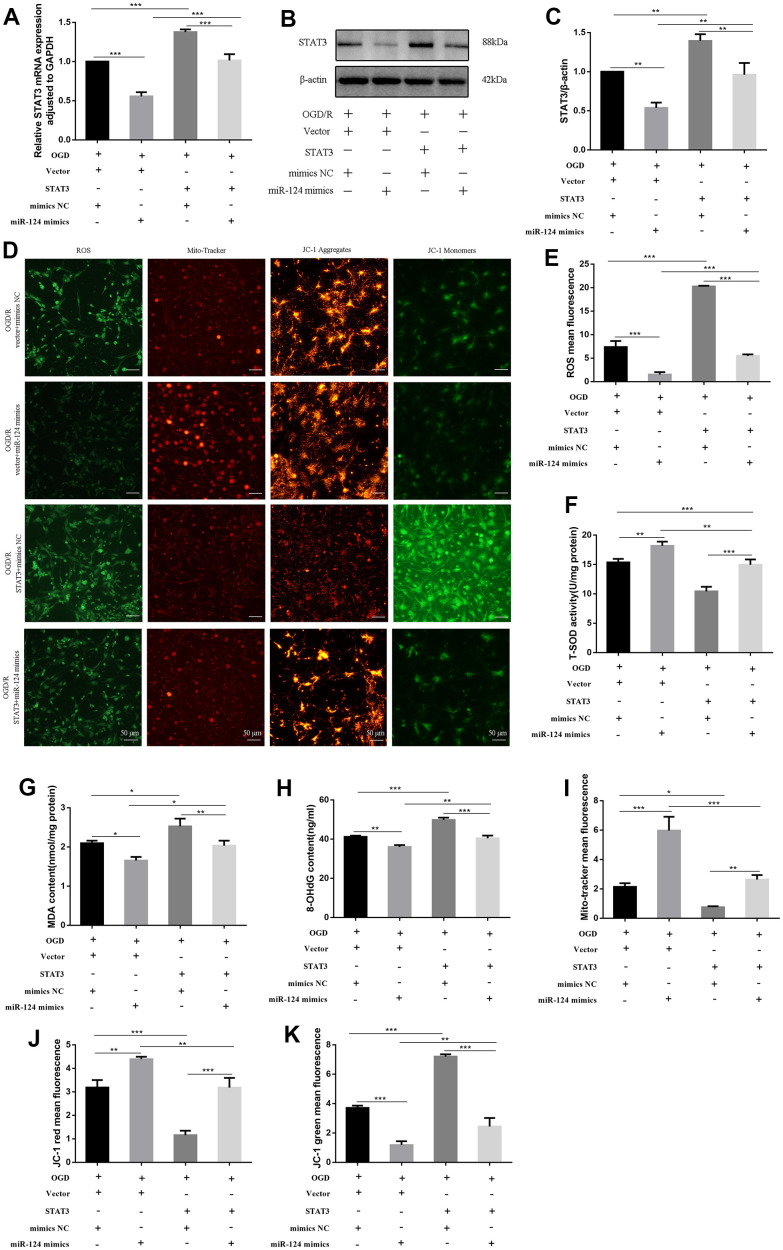
***STAT3* overexpression promotes oxidative stress in OGD-treated neurons.** The neurons were transfected with vector, *STAT3*(ORF), or co-transfected with vector and mimics NC, or co-transfected with vector and *miR-124* mimics, or co-transfected with *STAT3*(ORF) and mimics NC, or co-transfected with *STAT3*(ORF) and miR-*124* mimics. (**A**) The *STAT3* mRNA was detected by qRT-PCR; (**B**) The protein levels of STAT3 were detected by Western blot; (**C**) Quantitative analysis of the protein levels of STAT3; (**D**) Intracellular ROS was measured using DCFH-DA with a fluorescence microscope; Representative photomicrograph of mito-tracker red; Representative photomicrographs of fluorescence shift from red to green of JC-1 staining. Scale bar, 50μm; (**E**) Quantitative analysis green fluorescence of ROS; (**F**) The SOD activity was determined by the commercial kits; (**G**) The content of MDA was determined by the commercial kits; (**H**) The content of 8-OHdG was determined by the commercial kits; (**I**) Quantitative analysis of mito-tracker red; (**J**, **K**) Quantitative analysis of fluorescence shift from red to green of JC-1 staining; Data were expressed as mean ± SD (derived from three independent experiments for each sample). NS, not significant for *p* > 0.05, * *p* < 0.05, ** *p* < 0.01, and *** *p* < 0.001 (one-way analysis of variance with Tukey’s post hoc tests).

We further validated this interaction by constructing *STAT3* (open reading frame [ORF]) and co-transfecting it with *miR-124* mimics in the OGD/R model. Both the mRNA and protein levels of *STAT3* were significantly higher in the *STAT3* (ORF) + mimics NC group compared to the vector + mimics NC group (*p* < 0.05, Figure 4A–4C). In comparison to the vector + *miR-124* mimics group, the levels of ROS, MDA, and 8-OHdG were markedly elevated, and the activity of SOD was inhibited in the *STAT3* (ORF) + mimics NC group (*p* < 0.05, Figure 4D–4H). The mito-tracker red fluorescence levels marking live cells in the *STAT3* (ORF) + mimics NC group were lower than in the vector + *miR-124* mimics group (*p* < 0.05, Figure 4D, 4I). Additionally, in the *STAT3* (ORF) + mimics NC group, the red fluorescence decreased, and the green fluorescence increased compared to the vector + *miR-124* mimics group, indicating MMP loss (*p* < 0.05, Figure 4D, 4J, 4K). In summary, the protective effect of *miR-124* mimics after OGD/R was significantly diminished by *STAT3* overexpression.

### Knockdown of *STAT3* reverses the effects of *miR-124* inhibitor on oxidative stress in OGD/R-treated neurons

**Figure 5:**
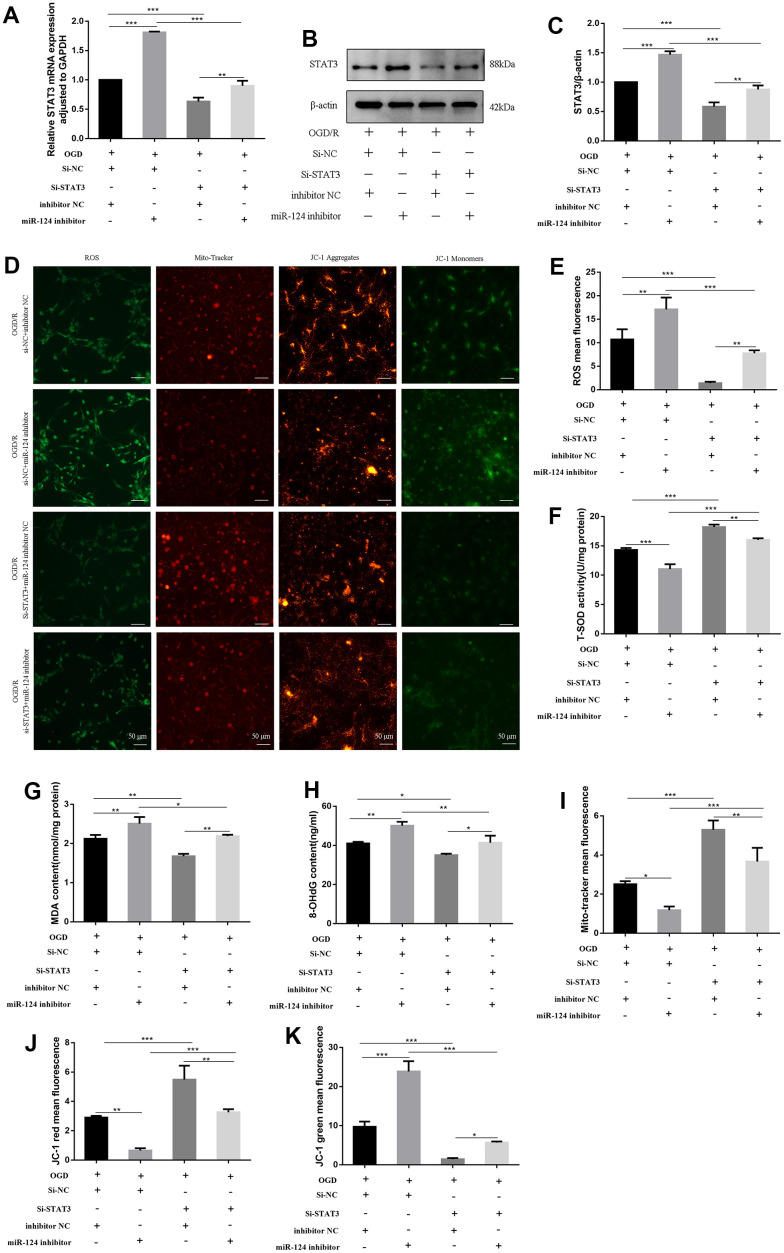
**Knockdown of *STAT3* reversed the effects of *miR-124* inhibitor on oxidative stress in OGD-treated neurons.** The neurons were transfected with si-NC, si-*STAT3*, or co-transfected with si-NC and inhibitor NC, or co-transfected with si-NC and *miR-124* inhibitor, or co-transfected with si-*STAT3* and inhibitor NC, or co-transfected with si-*STAT3* and *miR-124* inhibitor. (**A**) The *STAT3* mRNA was detected by qRT-PCR; (**B**) The protein levels of STAT3 were detected by Western blot; (**C**) Quantitative analysis of the protein levels of STAT3; (**D**) Intracellular ROS was measured using DCFH-DA with a fluorescence microscope; Representative photomicrograph of mito-tracker red; Representative photomicrographs of fluorescence shift from red to green of JC-1 staining. Scale bar, 50μm; (**E**) Quantitative analysis green fluorescence of ROS; (**F**) The SOD activity was determined by the commercial kits; (**G**) The content of MDA was determined by the commercial kits; (**H**) The content of 8-OHdG was determined by the commercial kits; (**I**) Quantitative analysis of mitochondria tracker red; (**J**, **K**) Quantitative analysis of fluorescence shift from red to green of JC-1 staining; Data were expressed as mean ± SD (derived from three independent experiments for each sample). NS, not significant for *p* > 0.05, * *p* < 0.05, ** *p* < 0.01, and *** *p* < 0.001 (one-way analysis of variance with Tukey’s post hoc tests).

We employed small interfering RNAs (siRNAs) to knock down *STAT3* expression and co-transfected *miR-124* inhibitor with si-*STAT3*. The levels of *STAT3* mRNA and protein in the si-*STAT3* + inhibitor NC group were significantly reduced compared to the si-NC + inhibitor NC group (*p* < 0.05, Figure 5A–5C). In the si-*STAT3* + *miR-124* inhibitor group, ROS, MDA, and 8-OHdG levels were significantly decreased, while SOD activity was enhanced compared to the si-NC + *miR-124* inhibitor group (*p* < 0.05, Figure 5D–5H). The mito-tracker red fluorescence levels marking live cells in the si-*STAT3* + *miR-124* inhibitor group were higher than in the si-NC + *miR-124* inhibitor group (*p* < 0.05, Figure 5D, 5I). Concurrently, in the si-*STAT3* + *miR-124* inhibitor group, the red fluorescence increased, and the green fluorescence decreased compared to the si-NC + *miR-124* inhibitor group, indicating an improvement in MMP (*p* < 0.05, Figure 5D, 5J, 5K). These findings suggest that the transfection of si-*STAT3* eliminated the adverse impact of *miR-124* inhibitor on oxidative stress.

### *MiR-124* attenuates the brain infarct volume, brain oedema, and neuronal damage, while improving the neurological reflex behavior of rats with HIBD *in vivo*

**Figure 6:**
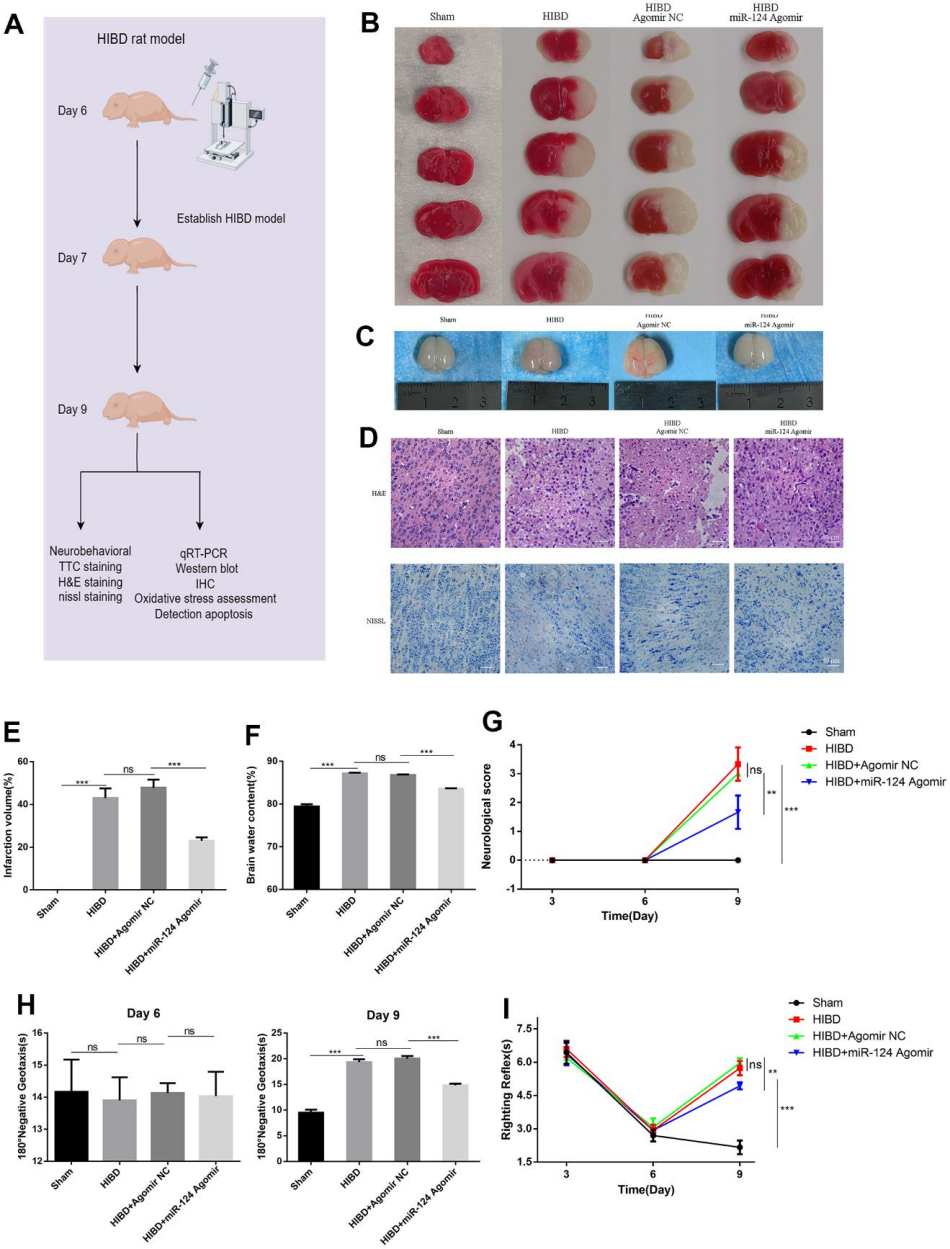
***MiR-124* attenuates the brain infarct volume, brain oedema, and neuronal damage, while improving the neurological reflex behavior of rats with HIBD *in vivo*.** (**A**) Overview of animal experiments procedures in the HIBD rat model; (**B**) Representative TTC stained coronal brain sections of each group (number of animals: n=5/group); (**C**) Representative images of newborn SD rat brain taken by digital camera after HI injury (number of animals: n=5/group); (**D**) Nissl staining and H&E staining of cerebral cortex region in each group(number of animals: n=5/group). Scale bar, 50μm; (**E**) Quantitative analysis of ipsilateral cerebral infarction volume in each group; (**F**) Quantitative analysis of brain water content; (**G**) Neurological score (number of animals: n=10/group); (**H**) Righting reflex times (number of animals: n=10/group); (**I**) Negative geotaxis reflex times (number of animals: n=10/group); Data are expressed as the mean ± SD. P < 0.05. NS, not significant for *p* > 0.05, * *p* < 0.05, ** *p* < 0.01, and *** *p* < 0.001 (one-way analysis of variance with Tukey’s post hoc tests).

To investigate the *in vivo* function of *miR-124*, we constructed a newborn HIBD rat model (Figure 6A). The cerebral infarction volume in HIBD neonatal rats was significantly larger compared to the control group. However, the area of cerebral infarct was smaller in the *miR-124* agomir group compared to the agomir NC group (*p* < 0.05, Figure 6B, 6E). In the HIBD group, oedema was visibly present in the ipsilateral cerebral hemisphere, as indicated in Figure 6C, 6F. In contrast, *miR-124* attenuated brain water content compared to the agomir NC group (*p* < 0.05). As shown in Figure 6D, neurons in the sham group were neatly arranged and tight, with clear and intact Nissl bodies. However, neurons in the HIBD group exhibited disorganization, nuclear shrinkage, widespread voids, and oedema between cells. Few remaining Nissl bodies were deeply stained and grainy. *MiR-124* agomir significantly reduced neuronal damage caused by HI damage compared to the agomir NC in newborn rats. Early neurological behavioral examinations were conducted on post-natal day 6 (1 h before intracerebroventricular injection) to establish baseline measures. The Zea-longa scores were determined to measure rat brain function. The HIBD group scored higher than the sham group, but the scores noticeably decreased with *miR-124* agomir compared to the agomir NC group on day 9 (*p* < 0.05, Figure 6G). Treatment for HIBD increased the mean times for both the 180° negative geotaxis reaction and the righting reflex on day 9. Compared to the agomir NC group, *miR-124* agomir significantly decreased righting reflex and negative reflex time in neonatal rats, indicating an improvement in short-term neurobehavioral impairment on day 9 (*p* < 0.05, Figure 6H, 6I). These results were consistent with the *in vitro* study, suggesting that *miR-124* protected HIBD rats against brain damage.

### *MiR-124* relieves oxidative stress and apoptosis in HIBD rats by inhibiting *STAT3 in vivo*

**Figure 7:**
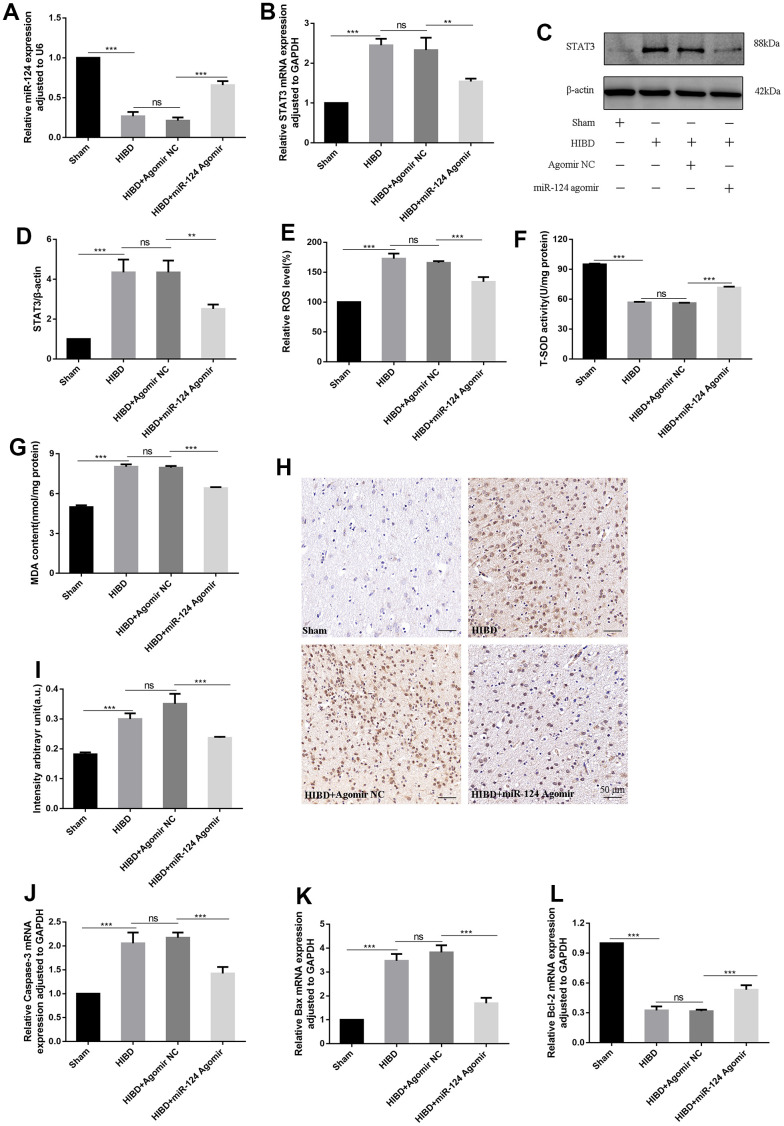
***MiR-124* relieves oxidative stress and apoptosis in HIBD rats by inhibiting *STAT3 in vivo*.** (**A**) The *miR-124* was detected by qRT-PCR; (**B**) The *STAT3* mRNA was detected by qRT-PCR; (**C**) The protein levels of STAT3 was detected by Western blot; (**D**) Quantitative analysis of the protein levels of STAT3; (**E**) The levels of intracellular ROS was measured; (**F**) The SOD activity was determined by the commercial kits; (**G**) The content of MDA was determined by the commercial kits; (**H**) Immunohistochemistry analysis of 8-OHdG intensity arbitrary in the peri-infarction cortex(number of animals: n=5/group). Scale bar, 50μm; (**I**) Quantitative analysis of protein levels of 8-OHdG by immunohistochemistry assay; (**J**–**L**) The mRNA levels of *Caspase*-3, *Bax*, and *Bcl-2* were detected by qRT-PCR. Data were expressed as mean ± SD (number of animals: n =5/group); NS, not significant for *p* > 0.05, * *p* < 0.05, ** *p* < 0.01, and *** *p* < 0.001 (one-way analysis of variance with Tukey’s post hoc tests).

To investigate how *miR-124* enhances nerve protection against brain damage in rat pups, we employed qRT-PCR and western blotting. The qRT-PCR results revealed a significant reduction in *miR-124* levels and an increase in *STAT3* mRNA levels in the HIBD group compared to the sham group (*p* < 0.05). Conversely, in the HIBD + *miR-124* agomir group, the *miR-124* level was higher, and *STAT3* mRNA levels were lower compared to the HIBD + agomir NC group (*p* < 0.05, Figure 7A, 7B). As shown in Figure 7C, 7D, the protein contents of STAT3 were significantly upregulated in the HIBD group compared to the sham group. Meanwhile, the overexpression of *miR-124* downregulated STAT3 protein levels in the HIBD + *miR-124* agomir group compared to the HIBD + agomir NC group *(p* < 0.05). These results suggest that *miR-124* exerts its protective effect on HIBD rats by regulating STAT3*.* We also assessed oxidative stress markers, including ROS, MDA, SOD, and 8-OHdG. In the HIBD group, the levels of ROS and MDA were higher, and the activity of SOD was decreased compared to the sham group. *MiR-124* overexpression significantly decreased the levels of ROS and MDA, while increasing SOD activity in the HIBD + *miR-124* agomir group compared to the HIBD + agomir NC group (*p* < 0.05, Figure 7E–7G). Immunohistochemistry (IHC) results showed that *miR-124* attenuates DNA oxidation in HIBD-induced rats. Compared with the sham group, the IHC staining of 8-OHdG increased in the HIBD group (*p* < 0.05). *MiR-124* agomir markedly reduced the IHC staining of 8-OHdG when comparing the HIBD + *miR-124* agomir group to the HIBD + agomir NC group (*p* < 0.05, Figure 7H, 7I). The data demonstrated that *miR-124* agomir could alleviate oxidative stress in HIBD rats. Finally, we analyzed the mRNA levels of apoptosis markers. The qRT-PCR results revealed higher mRNA levels of *Caspase-3* and *Bax* and lower mRNA levels of *Bcl-2* in the HIBD group than in the sham group. The *Caspase-3* and *Bax* mRNA levels were significantly lower, while the *Bcl-2* mRNA was higher in the HIBD + *miR-124* agomir group compared to the HIBD + agomir NC group (*p* < 0.05, Figure 7J–7L). In conclusion, *miR-124* may alleviate oxidative stress and apoptosis in HIBD rats by inhibiting *STAT3*.

**Figure 8:**
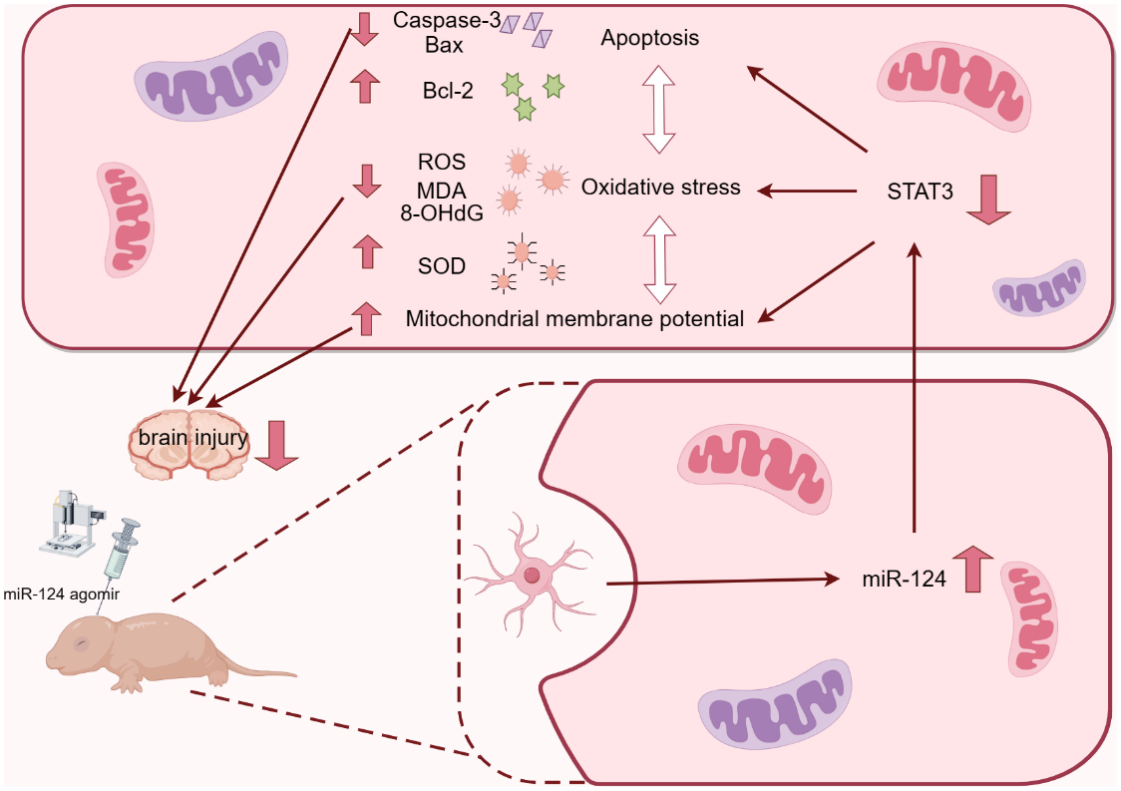
Graphical Abstract. A Graphical abstract of *miR-124* relieved oxidative stress and apoptosis on neonatal hypoxic–ischaemic brain damage.

## DISCUSSION

HIE affects ~1.5 of every 1,000 newborns, posing a significant risk of permanent neurological impairment [20]. The consequences of HIE can have a profound impact on affected families and extend to broader societal implications. TH has proven beneficial for newborns with moderate to severe HIE, the therapeutic window is short, and current protocols are only partially effective, prompting the search for new, safe, and effective treatments [21]. Previous research has highlighted the crucial role of *miR-124* in brain development, and abnormal *miR-124* expression has been associated with nervous system diseases [22–25]. Therefore, our experiments aimed to investigate the causal nature of the interaction between *miR-124* and HIBD. In our research, we observed a significant decrease in *miR-124* levels in OGD/R-induced primary cortical neurons. This aligns with a clinical study which have shown decreased levels of *miR-124* in patients with acute cerebral infarction, accompanied by increased levels of IL-6, IL-8, and CRP, with a significant negative correlation [26]. Furthermore, the upregulation of *miR-124* significantly alleviated oxidative stress, apoptosis, and mitochondrial impairment in cells following OGD/R treatment. Lastly, we propose that *miR-124* exerts a neuroprotective effect in HIBD by regulating *STAT3*. To our knowledge, this study is the first to explore how *miR-124* targets *STAT3* to inhibit oxidative stress and apoptosis in HIBD.

Oxidative stress plays a crucial role in the development of HIBD, especially given the vulnerability of the newborn brain to hypoxia due to incomplete development. Previous research has indicated that hypoxia-ischemia triggers oxidative stress, leading to increased formation of ROS and decreased antioxidant enzyme SOD activity in the brain, ultimately resulting in brain damage [27–33]. Our data revealed a noticeable increase in ROS DCF fluorescence intensity after OGD/R treatment, but this phenomenon was reversed by *miR-124* mimics. ROS serves as a key molecular regulator in signal pathways and biological functions. Excessive ROS production can cause permanent oxidative reactions, damaging proteins, DNA (marked by 8-OHdG, a DNA oxidative damage marker), lipids (marked by MDA, a lipid peroxidation product), and more, ultimately leading to cell death [34–36]. Therefore, we assessed other markers of oxidative stress in our study. The results indicated that *miR-124* overexpression promoted SOD activity while reducing levels of MDA and 8-OHdG in OGD-treated neurons, suggesting that *miR-124* could alleviate oxidative stress. This finding aligns with Silva’s research, highlighting the potential of *miR-124* as a therapeutic strategy in HIBD by maintaining a balance between promoting and inhibiting oxidative stress [37]. *In vivo* studies further supported these results, showing that upregulation of *miR-124* level reduced the incidence of cerebral infarction and brain oedema, alleviating neurological impairments and oxidative stress.

During the initial phase of hypoxia-ischemia, cells swell, and excess excitatory amino acids are produced, leading to oxidative stress and the generation of numerous free radicals, especially hydroxyl radicals (•OH−). These radicals mediate neuronal impairment through various mechanisms, including the accumulation of 8-OHdG and MDA, as well as mitochondrial dysfunction [38]. MMP is crucial for membrane stability, and our study used mito-tracker red fluorescent probe staining and JC-1 assay to evaluate mitochondrial-specific markers in OGD/R-treated neurons. The results demonstrated a relationship between *miR-124* and mitochondrial impairment in HIBD. *MiR-124* overexpression improved mitochondrial damage, as indicated by mito-tracker red labeling, and stabilized mitochondrial membrane potential. As part of the apoptotic pathway activation, the release of cytochrome c from mitochondria is a critical stage [39, 40]. The diffusion of cytochrome c and pro-apoptotic proteins triggers the activation of Caspase-3 [29]. Bax and Bcl-2 are commonly used to assess the severity of apoptosis. Our data showed that *miR-124* mimics significantly reduced mRNA levels of *Caspase-3* and *Bax*, while increasing mRNA levels of *Bcl-2* from a molecular perspective. This aligns with previous research confirming the impact of oxidative stress on neuronal apoptosis in HIBD, indicating that *miR-124* overexpression suppresses oxidative stress, alleviating mitochondrial dysfunction and apoptosis in neurons after OGD/R treatment [41].

The neuroprotective effect of *miR-124* prompted us to delve into the underlying mechanisms of HIBD. We used bioinformatics methods and a dual luciferase reporter system to analyze the interaction between *miR-124* and *STAT3*. *STAT3*, a redox-regulated protein belonging to the STAT family, remains latent in the cytoplasm of resting cells until activated by growth factors and cytokines binding to specific cell receptors [42]. It plays a role in diverse biological outcomes, including cell proliferation, differentiation, apoptosis, inflammation, modulation of the immune system, and tumorigenesis. While the classical pathway involves the transfer of STAT3 from the cytoplasm to the nucleus, recent findings indicate non-canonical roles for STAT3 outside the nucleus, regulating mitochondrial functions, endoplasmic reticulum, and lysosomes [43]. Numerous studies have established a strong relationship between STAT3 and oxidative stress. Research by Zhu highlighted the protective role of regulating the *IL-6/STAT3* pathway against oxidative stress in brain occlusion/reperfusion damage [44]. Huo demonstrated that inhibiting the *IL-6/STAT3* pathway could exert cardioprotective effects by mitigating oxidative stress-induced mitochondrial dysfunction in a heart failure model [45]. Additionally, studies by Zhao and Mehla showed that *p-STAT3* deficiency could reduce inflammation, oxidative stress, and proliferation in liver damage, while inhibiting *STAT3* could enhance cognitive function, functional connectivity, and cerebral blood flow in Alzheimer’s disease by reducing neuritic plaques, cerebral amyloid angiopathy, oxidative stress, and neuroinflammation [46, 47]. Consistent with prior research, our HIBD rat models exhibited increased levels of both *STAT3* mRNA and protein [48]. Furthermore, we observed a negative interaction between *miR-124* and *STAT3*. Using *STAT3* (ORF) and si-*STAT3*, we further analyzed how *miR-124* affects *STAT3.* The results showed that *STAT3* (ORF) and si-*STAT3* could counteract the roles of *miR-124* mimics or inhibitor in neurons after OGD/R treatment. In conclusion, our study suggests that *miR-124* may exert its neuroprotective role in HIBD by inhibiting *STAT3*.

A previous study demonstrated that *miR-124* promotes neuronal survival and inhibits apoptosis in HIBD, but the underlying mechanism was not explored [49]. While abnormal expressions of *miR-124* and *STAT3* have been implicated in various nervous system diseases, the inhibition of oxidative stress and apoptosis by *miR-124* targeting *STAT3* in HIBD has not been reported [50–53]. Nevertheless, our experiment has some limitations. We focused solely on cortical neurons, neglecting other brain areas such as hippocampal neurons, and we intend to address this limitation in future studies. Additionally, the long-term prognosis effects of *miR-124* in HIBD, including athletic and learning abilities, remain unclear and warrant further investigation. The translation of effective miRNA therapy from experiments to clinical application is an ongoing challenge. Although our research provides a rationale for the potential therapeutic role of *miR-124*, further studies are needed to obtain direct evidence of the in-depth mechanism and assess long-term effects in clinical samples.

In summary, our findings indicate that *miR-124* alleviates oxidative stress and apoptosis by directly targeting *STAT3* in both *in vivo* and *in vitro* analyses. This suggests that *miR-124* holds significant potential for addressing HIBD and may serve as a solid foundation for future treatments of this condition.

## MATERIALS AND METHODS

### Primary cortical neurons culturing

Primary cortical neurons were obtained using Viesselman’s method, with all procedures conducted on ice [54]. Brains from Sprague-Dawley rats were rapidly isolated within 24 h postnatally and cut into ~1 mm^3^ pieces using ophthalmic scissors. The brain, following isolation, was washed with Dulbecco’s modified Eagle Medium (DMEM/F12, 10% fetal bovine serum [FBS] + 100 μg/mL penicillin and streptomycin) after exposure to 0.25% trypsin at 37° C for 15 min. After centrifugation (1,500 rpm, 5 min), single-cell suspension was created by resuspending in a fresh complete culture medium and filtering. Culture plates were coated with poly-d-lysine, and neurons were then seeded at a density of ~2 × 10^5^/mL for 4 h at 37° C with 5% CO_2_. A neuron-specific medium (neurobasal medium + 2% B27, without serum; Gibco, Billings, MT, USA), was utilized instead of the complete medium. The medium was refreshed every 3 days, and anti-Map-2 immunofluorescence staining was employed to assess the purity of cultured neurons up to day 7.

### Immunofluorescence staining

The cell slides were blocked for 1 h in 0.1 M phosphate-buffered saline (PBS) with 5% FBS and 0.3% Triton X at 25° C. Following the blocking, the slides were washed and then exposed to 50 μL rabbit anti-Map-2 antibody (AF4081, 1:200; Affinity Biosciences) overnight at 4° C. Subsequently, the cell slides were triple-rinsed with PBS, and each slide received 50–100 μL fluorescein isothiocyanate secondary antibodies for 1 h. Finally, DAPI was incubated on the cell slides in a dark room for 10 min. Images were captured using a fluorescent microscope, and the quantity of Map-2-positive cells was determined using ImageJ software.

### Establishment of an OGD/R model of primary cortical neurons

The primary cortical neurons were cultured until day 7, and after the OGD/R treatment, the primary cortical neurons were used to simulate *in vitro* HIBD. Following the substitution of glucose-deprived Neurobasal-A medium for the regular medium, the neurons were cultured in a hypoxic environment (1% O_2_, 5% CO_2_, and 94% N_2_) for 2 h at 37° C. Subsequently, the glucose-deprived Neurobasal-A medium was replaced with a neuron-specific medium, and the cells were incubated for 12 h in a standard incubator (37° C, 5% CO_2_, and 95% air). The normal group consisted of neurons that did not undergo OGD/R treatment.

### Cell transfection

miRNA mimics, miRNA inhibitor, STAT3 siRNA, and their corresponding negative controls (mimics-NC, inhibitor-NC, and si-NC) were synthesized by Ribobio (Guangzhou, China). Transfections in neurons were conducted using either the CP reagent (Ribobio, Guangzhou, China) or Lipofectamine 2000 reagent (Invitrogen, Carlsbad, CA, USA), following the manufacturer’s protocol.

### Cell viability assay

Cell viability was assessed using the Cell Counting Kit 8 (C0037; Beyotime, Shanghai, China). In 96-well plates, 20 μL CCK-8 solution was added to each well, and neurons were then seeded at a density of 1 × 10^4^ cells/well. After a 2-hour incubation in a 37° C incubator, the absorbance for each well was recorded at 450 nm.

### RNA isolation, complementary DNA (cDNA) synthesis, and RT-qPCR

The total RNA was extracted using the Trizol reagent (Invitrogen) following the manufacturer’s protocol. The Reverse Transcription Kit (RR037A; Takara, Dalian, China) or the Bulge-Loop miRNA qRT-PCR starter kit (C10211; Ribobio) was employed for reverse-transcribing the RNA into cDNA. Primer sequences are detailed in Table 1. Each sample was subjected to three technical replicates, and calculations were performed utilizing a relative quantitative technique, specifically the 2^–ΔΔCt^ method.

**Table 1:** Primer sequences.

**Primers RT-PCR**	**Primers (5′–3′)**	
	Forward	Reverse
*NEUROD1* *LAMC1* *ITGB1* *KLF15* *NR3C1* *CAV1* *STAT3*	GACGGGGTCCCAAAAAGAAAA AACTGCCACTGACATCAGAGT TGACCCCAATACCAATCTCCC CAGAGAGCGTCAAGGTCGC GACTCCAAAGAATCCTTAGCTCC GCGACCCCAAGCATCTCAA CACCTTGGATTGAGAGTCAAGAC	GCCAAGCGCAGTGTCTCTATT ACTTTGGGGTCGTTAAACACTTC CCTGAAGTGAACTTGTGGCAG TTCGCACAAACTTTGAGGGCA CTCCACCCCTCAGGGTTTTAT ATGCCGTCGAAACTGTGTGT AGGAATCGGCTATATTGCTGGT
*Bcl-2*	GCTACCGTCGTGACTTCGC	CCCCACCGAACTCAAAGAAGG
*Bax*	AGACAGGGGCCTTTTTGCTAC	AA TTCGCCGGAGACACTCG
*Caspase-3*	CTCGCTCTGGTACGGA TGTG	TCCCA TAAA TGACCCCTTCA TCA
*GAPDH*	CGACTTCAACAGCAACTCCCACTCTTCC	TGGGTGGTCCAGGGTTTCTTACTCCTT

### Protein isolation and Western blot

The NP-40 lysis buffer (P0013F; Beyotime) was utilized, following the manufacturer’s protocol, to extract total protein from both the brains of neonatal rats and primary cortical neurons. In brief, the brains or neurons were immersed in the lysis buffer, and an ultrasonic processor was employed to disrupt the cerebral homogenate of rats or neurons. The BCA protein assay kit (P0010; Beyotime) was employed to determine the protein concentration. Subsequently, the protein samples were boiled at 10° C for 10 min and stored at –80° C. Following separation by 10% sodium dodecyl sulfate-polyacrylamide gel, the proteins were transferred onto nitrocellulose membranes (GE Healthcare, Chicago, IL, USA). The nitrocellulose membranes were then blocked with 5% BSA in Tris-buffered saline with Tween 20 (TBS-T) at 25° C for 1 h. Primary antibodies, specifically rabbit anti-STAT3 (AF6294, 1:500; Affinity Biosciences) and rabbit anti-*β*-Actin (81115-1-RR, 1:5,000; Proteintech), were applied to the nitrocellulose membranes overnight at 4° C. On the following day, after the membranes were thrice washed with TBS-T, they were incubated at 25° C with corresponding secondary antibodies coupled to horseradish peroxidase for 1 h. The ImageJ software was employed for semi-quantification of the protein band intensities, and each experiment was carried out in triplicate.

### Dual-luciferase reporter gene assay

The bioinformatics websites miRDB (http://mirdb.org/), miRTarBase (http://mirtarbase.cuhk.edu.cn/php/index.php), and TargetScan (http://www.targetscan.org/vert_72/) were employed to predict potential target genes of *miR-124*. The intersection of their predictions was used to identify *STAT3* as a potential *miR-124* target gene. TargetScan was utilized to predict the binding sites for *STAT3* and *miR-124*. In HEK293T cells, Lipofectamine 2000 was used to co-transfect WT or MUT *STAT3* type luciferase reporter plasmid vectors with *miR-124*-mimic or NC-mimic. The Dual-Luciferase Reporter Assay Kit (Promega, Madison, WI, USA) was used to measure renilla and firefly luciferase readings after harvesting cells 48 h post-transfection.

### Assessment of oxidative stress

ROS production was assessed using the DCFH-DA probe (S0033; Beyotime). Neurons were treated with pre-formulated DCFH-DA in the dark for 20 min at 37° C. Subsequently, the medium was removed, and neurons were rinsed thrice with a serum-free culture medium. The optical density (OD) values were either measured or captured using a fluorescence microscope. The content of 8-OHdG and MDA, as well as the activity of SOD, were determined using assay kits from Nanjing Jiancheng (Nanjing, China).

### Detection of mitochondrial impairment

Mitochondrial-specific markers of living cells were identified using the Mito-Tracker Red fluorescent probe stain method, and the JC-1 assay was employed to assess the MMP in the OGD/R-treated cell model. After neuronal OGD/R, each well was treated with Mito-Tracker Red working fluid (C1032; Beyotime) instead of the culture medium in a dark incubator at 37° C for 30 min. Subsequently, the Mito-Tracker Red working fluid was replaced with fresh cell culture at 37° C, and images were captured using a fluorescent microscope. The mitochondrial red fluorescence staining was quantified using ImageJ software. For the JC-1 assay, after neuronal OGD/R, each well was treated with a JC-1 working solution (C2006; Beyotime) instead of the culture medium for 20 min in a dark incubator at 37° C. Following this, JC-1 dye buffer was used to wash neurons three times, and images were captured with a fluorescent microscope. The shift from red (aggregates) to green (monomer) fluorescence indicated a decrease in MMP. Changes in JC-1 red and JC-1 green fluorescence intensity were calculated using ImageJ software.

### Animals and HIBD rat model

Pregnant Sprague-Dawley rats were provided by the Animal Experimental Center of Huazhong University of Science and Technology. The rats were housed in specific-pathogen-free environments with a fixed light–dark cycle, maintained at 25±1° C with 55–60% relative humidity, and provided free access to food and water. To induce HIBD, rats weighing 15–18 g at 1 week after birth were used, following the Rice–Vannucci method [55]. Anesthesia induction involved 3% isoflurane, and maintenance dosing was 1.5% isoflurane in newborn rats. The left common carotid artery was separated and ligated, and the skin and subcutaneous tissue were sutured. After a 1.5-hour rest with the mother, the newborn rats were exposed to warm conditions for 2 h (37° C, 8% O_2_, and 92% N_2_). The sham group underwent general anesthesia only for vessel separation. All procedures were performed under general anesthesia to minimize pain, maintain strict aseptic conditions, and ensure the warmth of neonatal rats. A total of 80 neonatal rats of both sexes were randomly assigned to four groups, each consisting of 20 rats: **(**1) sham group, (2) HIBD group, (3) HIBD + agomir NC group, and (4) HIBD + *miR-124* agomir group.

### Intracerebroventricular injection

The newborn rats were anesthetized and subsequently placed into a stereotactic frame. A 10 μL needle (Hamilton, Reno, NV, USA) was then inserted into the specified injection coordinates (2 mm in front of the posterior fontanelle, 1.5 mm to the side of the midline, and 3 mm below the skull surface). The *miR-124* agomir and agomir NC (Ribobio, Guangzhou, China) were slowly injected (2 μL each, over 5 min) into the left lateral ventricle of the rat brain, administered once prior to the 24-hour HIBD treatment for total one time. The needle was left in place for 10 min before being withdrawn to prevent liquid reflux. The dosage of miRNA was determined based on previous studies [56–58].

### Neurological deficit score and early behavioral examination

As previously mentioned, neurological deficits were assessed using the Zea-longa score on post-natal day 3 (day 3), post-natal day 6 (day 6, 1 h before intracerebroventricular injection), and post-natal day 9 (day 9, 48 h after the HIBD model was established) [59, 60]. The scoring criteria were as follows: 0, no symptoms of neurological deficits; 1, left forepaw paresis; 2, rats turning to the ischemic side during crawling; 3, rats rolling to the ischemic side during crawling; and 4, inability to crawl spontaneously or loss of consciousness. The scores exhibited a positive correlation with brain damage, and a successful mold was duplicated with a score between 2 and 4. Early behavioral examinations were conducted using a double-blind method. The righting reflex time after placing neonatal rats on their backs was recorded on day 3, day 6, and day 9. Each rat’s average value was recorded three times, with a maximum test duration of 30 s. On days 6 and 9, rats were placed on inclined planes tilted at 45° to assess negative geotaxis reflex. The time taken to rotate 180° and turn its head upward was recorded for each rat, with a maximum permitted test duration of 120 s. All assessments were evaluated and calculated by three professionals blinded to the experimental design.

### Infarct area measurements and brain water content

The brain infarct volume was determined through 2,3,5-triphenyltetrazolium chloride (TTC) staining. Neonatal rat brains were rapidly extracted on ice after inhalation anesthesia and kept in a -20° C refrigerator for ~15 min. Subsequently, the brains were sliced into 2-mm-thick coronal planes, submerged in TTC staining solution, and stained for 30 min at 37° C in a dark incubator. The planes were rotated every 10 min during staining to ensure even exposure to the dye. After staining, the brain sections were submerged in 4% paraformaldehyde overnight at 4° C. The TTC staining results showed result revealed that normal brains were consistently red, while yet the infarcted area appeared of infarction was white. The cerebral infarct area was measured using ImageJ software. The infarct Volume percentage was calculated using the formula: Infarct volume (%) = [(the contralateral hemisphere area – non-infarcted ipsilateral hemisphere area) / 2 × (the contralateral hemisphere area)] × 100%. Following the measurement of the left cerebral hemisphere weight, the brain was dehydrated for 24 h at 80° C, and the weight of the dry brain was recorded. Brain water content was calculated using the formula: Brain water content (%) = [(the weight of wet brain – the weight of dry brain) / the weight of wet brain] × 100%.

### Hematoxylin and eosin (H&E) staining and Nissl staining

The neuronal pathological changes in neonatal rats with HIBD were assessed using H&E and Nissl staining staining. The brains from each experimental group were extracted and perfused with cold PBS followed by a 4% paraformaldehyde solution. After overnight external fixation in a 4% paraformaldehyde solution, the brains underwent dehydration using sucrose solutions of varying concentrations. Subsequently, the brains were embedded in paraffin and sliced into 5-μm thick coronal sections for H&E staining, Nissl staining, and IHC. Standard protocols were employed for H&E staining and Nissl staining on the coronal brain sections, and images were captured using a light microscope.

### Immunohistochemistry (IHC)

The paraffin-embedded sections were obtained following the procedure outlined for H&E staining. Samples were incubated with rabbit anti-8-OhdG antibody (bs-1278R, 1:200; Bioss) overnight at 4° C and then rinsed three times with PBS. Subsequently, a secondary antibody was applied and incubated with samples at room temperature for 30 min. A reaction with diaminobenzidine resulted in a positive signal indicated by a tan color of the target protein. Images were captured using a light microscope, and the mean optical density value was computed using ImageJ software.

### Statistical analysis

All results were expressed as the mean ± standard deviation of three replicates for each experiment. Statistical analyses were conducted using GraphPad Prism v6.0. The comparison between two groups was assessed using the Student’s *t*-test. Differences among multiple groups were analyzed using one-way analysis of variance followed by Tukey’s post-hoc tests. A significance level of *p* < 0.05 was considered statistically significant.

### Availability of data and materials

The datasets used and/or analyzed during the current study available from the corresponding author on reasonable request.

## References

[1] Ponnusamy V, Yip PK. The role of microRNAs in newborn brain development and hypoxic ischaemic encephalopathy. Neuropharmacology. 2019; 149:55–65. 10.1016/j.neuropharm.2018.11.041 30716413

[2] Rodríguez M, Valez V, Cimarra C, Blasina F, Radi R. Hypoxic-Ischemic Encephalopathy and Mitochondrial Dysfunction: Facts, Unknowns, and Challenges. Antioxid Redox Signal. 2020; 33:247–62. 10.1089/ars.2020.8093 32295425

[3] Ferriero DM. Neonatal brain injury. N Engl J Med. 2004; 351:1985–95. 10.1056/NEJMra041996 15525724

[4] Ranjan AK, Gulati A. Advances in Therapies to Treat Neonatal Hypoxic-Ischemic Encephalopathy. J Clin Med. 2023; 12:6653. 10.3390/jcm12206653 37892791 PMC10607511

[5] Glass HC. Hypoxic-Ischemic Encephalopathy and Other Neonatal Encephalopathies. Continuum (Minneap Minn). 2018; 24:57–71. 10.1212/CON.0000000000000557 29432237

[6] Mota-Rojas D, Villanueva-García D, Solimano A, Muns R, Ibarra-Ríos D, Mota-Reyes A. Pathophysiology of Perinatal Asphyxia in Humans and Animal Models. Biomedicines. 2022; 10:347. 10.3390/biomedicines10020347 35203556 PMC8961792

[7] Lemyre B, Chau V. Hypothermia for newborns with hypoxic-ischemic encephalopathy. Paediatr Child Health. 2018; 23:285–91. 10.1093/pch/pxy028 30657134 PMC6007306

[8] Juul SE, Comstock BA, Heagerty PJ, Mayock DE, Goodman AM, Hauge S, Gonzalez F, Wu YW. High-Dose Erythropoietin for Asphyxia and Encephalopathy (HEAL): A Randomized Controlled Trial - Background, Aims, and Study Protocol. Neonatology. 2018; 113:331–8. 10.1159/000486820 29514165 PMC5980685

[9] Saliminejad K, Khorram Khorshid HR, Soleymani Fard S, Ghaffari SH. An overview of microRNAs: Biology, functions, therapeutics, and analysis methods. J Cell Physiol. 2019; 234:5451–65. 10.1002/jcp.27486 30471116

[10] Lagos-Quintana M, Rauhut R, Yalcin A, Meyer J, Lendeckel W, Tuschl T. Identification of tissue-specific microRNAs from mouse. Curr Biol. 2002; 12:735–9. 10.1016/s0960-9822(02)00809-6 12007417

[11] Ludwig N, Leidinger P, Becker K, Backes C, Fehlmann T, Pallasch C, Rheinheimer S, Meder B, Stähler C, Meese E, Keller A. Distribution of miRNA expression across human tissues. Nucleic Acids Res. 2016; 44:3865–77. 10.1093/nar/gkw116 26921406 PMC4856985

[12] Shi F, Yang Y, Wang T, Kouadir M, Zhao D, Hu S. Cellular Prion Protein Promotes Neuronal Differentiation of Adipose-Derived Stem Cells by Upregulating miRNA-124. J Mol Neurosci. 2016; 59:48–55. 10.1007/s12031-016-0733-8 26947028

[13] Mokabber H, Najafzadeh N, Mohammadzadeh Vardin M. miR-124 promotes neural differentiation in mouse bulge stem cells by repressing Ptbp1 and Sox9. J Cell Physiol. 2019; 234:8941–50. 10.1002/jcp.27563 30417370

[14] Xue Q, Yu C, Wang Y, Liu L, Zhang K, Fang C, Liu F, Bian G, Song B, Yang A, Ju G, Wang J. miR-9 and miR-124 synergistically affect regulation of dendritic branching via the AKT/GSK3β pathway by targeting Rap2a. Sci Rep. 2016; 6:26781. 10.1038/srep26781 27221778 PMC4879704

[15] Rajasethupathy P, Fiumara F, Sheridan R, Betel D, Puthanveettil SV, Russo JJ, Sander C, Tuschl T, Kandel E. Characterization of small RNAs in Aplysia reveals a role for miR-124 in constraining synaptic plasticity through CREB. Neuron. 2009; 63:803–17. 10.1016/j.neuron.2009.05.029 19778509 PMC2875683

[16] Periyasamy P, Liao K, Kook YH, Niu F, Callen SE, Guo ML, Buch S. Cocaine-Mediated Downregulation of miR-124 Activates Microglia by Targeting KLF4 and TLR4 Signaling. Mol Neurobiol. 2018; 55:3196–210. 10.1007/s12035-017-0584-5 28478506 PMC5673594

[17] Ke J, Zhao F, Luo Y, Deng F, Wu X. MiR-124 Negatively Regulated PARP1 to Alleviate Renal Ischemia-reperfusion Injury by Inhibiting TNFα/RIP1/RIP3 Pathway. Int J Biol Sci. 2021; 17:2099–111. 10.7150/ijbs.58163 34131409 PMC8193263

[18] Han F, Chen Q, Su J, Zheng A, Chen K, Sun S, Wu H, Jiang L, Xu X, Yang M, Yang F, Zhu J, Zhang L. MicroRNA-124 regulates cardiomyocyte apoptosis and myocardial infarction through targeting Dhcr24. J Mol Cell Cardiol. 2019; 132:178–88. 10.1016/j.yjmcc.2019.05.007 31100313

[19] Amruta N, Bix G. ATN-161 Ameliorates Ischemia/Reperfusion-induced Oxidative Stress, Fibro-inflammation, Mitochondrial damage, and Apoptosis-mediated Tight Junction Disruption in bEnd.3 Cells. Inflammation. 2021; 44:2377–94. 10.1007/s10753-021-01509-9 34420157 PMC8380192

[20] Li Y, Wisnowski JL, Chalak L, Mathur AM, McKinstry RC, Licona G, Mayock DE, Chang T, Van Meurs KP, Wu TW, Ahmad KA, Cornet MC, Rao R, et al. Mild hypoxic-ischemic encephalopathy (HIE): timing and pattern of MRI brain injury. Pediatr Res. 2022; 92:1731–6. 10.1038/s41390-022-02026-7 35354930 PMC9771796

[21] Wassink G, Davidson JO, Dhillon SK, Zhou K, Bennet L, Thoresen M, Gunn AJ. Therapeutic Hypothermia in Neonatal Hypoxic-Ischemic Encephalopathy. Curr Neurol Neurosci Rep. 2019; 19:2. 10.1007/s11910-019-0916-0 30637551

[22] Gu X, Xu X, Jia C, Wang J, Zhang J, Gao Q, Chen J. Molecular Mechanisms Involved in the Regulation of Neurodevelopment by miR-124. Mol Neurobiol. 2023; 60:3569–83. 10.1007/s12035-023-03271-5 36840845

[23] Sanuki R, Yamamura T. Tumor Suppressive Effects of miR-124 and Its Function in Neuronal Development. Int J Mol Sci. 2021; 22:5919. 10.3390/ijms22115919 34072894 PMC8198231

[24] Heiskanen M, Das Gupta S, Mills JD, van Vliet EA, Manninen E, Ciszek R, Andrade P, Puhakka N, Aronica E, Pitkänen A. Discovery and Validation of Circulating microRNAs as Biomarkers for Epileptogenesis after Experimental Traumatic Brain Injury-The EPITARGET Cohort. Int J Mol Sci. 2023; 24:2823. 10.3390/ijms24032823 36769143 PMC9918096

[25] Namkung H, Yukitake H, Fukudome D, Lee BJ, Tian M, Ursini G, Saito A, Lam S, Kannan S, Srivastava R, Niwa M, Sharma K, Zandi P, et al. The miR-124-AMPAR pathway connects polygenic risks with behavioral changes shared between schizophrenia and bipolar disorder. Neuron. 2023; 111:220–35.e9. 10.1016/j.neuron.2022.10.031 36379214 PMC10183200

[26] Zhou X, Qi L. miR-124 Is Downregulated in Serum of Acute Cerebral Infarct Patients and Shows Diagnostic and Prognostic Value. Clin Appl Thromb Hemost. 2021; 27:10760296211035446. 10.1177/10760296211035446 34702084 PMC8554555

[27] Alonso-Alconada D, Álvarez FJ, Goñi-de-Cerio F, Hilario E, Álvarez A. Cannabinoid-mediated Modulation of Oxidative Stress and Early Inflammatory Response after Hypoxia-Ischemia. Int J Mol Sci. 2020; 21:1283. 10.3390/ijms21041283 32074976 PMC7072925

[28] Auti A, Alessio N, Ballini A, Dioguardi M, Cantore S, Scacco S, Vitiello A, Quagliuolo L, Rinaldi B, Santacroce L, Di Domenico M, Boccellino M. Protective Effect of Resveratrol against Hypoxia-Induced Neural Oxidative Stress. J Pers Med. 2022; 12:1202. 10.3390/jpm12081202 35893296 PMC9330416

[29] Coimbra-Costa D, Alva N, Duran M, Carbonell T, Rama R. Oxidative stress and apoptosis after acute respiratory hypoxia and reoxygenation in rat brain. Redox Biol. 2017; 12:216–25. 10.1016/j.redox.2017.02.014 28259102 PMC5334548

[30] Ortiz GG, Pacheco Moisés FP, Mireles-Ramírez M, Flores-Alvarado LJ, González-Usigli H, Sánchez-González VJ, Sánchez-López AL, Sánchez-Romero L, Díaz-Barba EI, Santoscoy-Gutiérrez JF, Rivero-Moragrega P. Oxidative Stress: Love and Hate History in Central Nervous System. Adv Protein Chem Struct Biol. 2017; 108:1–31. 10.1016/bs.apcsb.2017.01.003 28427557

[31] Terraneo L, Paroni R, Bianciardi P, Giallongo T, Carelli S, Gorio A, Samaja M. Brain adaptation to hypoxia and hyperoxia in mice. Redox Biol. 2017; 11:12–20. 10.1016/j.redox.2016.10.018 27835780 PMC5107733

[32] Wang D, Li B, Wang S, Hao Y, Wang H, Sun W, Cao J, Zhou X, Zheng B. Engineered inhaled nanocatalytic therapy for ischemic cerebrovascular disease by inducing autophagy of abnormal mitochondria. NPJ Regen Med. 2023; 8:44. 10.1038/s41536-023-00315-1 37567914 PMC10421937

[33] Zhao M, Zhu P, Fujino M, Zhuang J, Guo H, Sheikh I, Zhao L, Li XK. Oxidative Stress in Hypoxic-Ischemic Encephalopathy: Molecular Mechanisms and Therapeutic Strategies. Int J Mol Sci. 2016; 17:2078. 10.3390/ijms17122078 27973415 PMC5187878

[34] Thangwong P, Jearjaroen P, Govitrapong P, Tocharus C, Tocharus J. Melatonin improves cognitive function by suppressing endoplasmic reticulum stress and promoting synaptic plasticity during chronic cerebral hypoperfusion in rats. Biochem Pharmacol. 2022; 198:114980. 10.1016/j.bcp.2022.114980 35219702

[35] Forrester SJ, Kikuchi DS, Hernandes MS, Xu Q, Griendling KK. Reactive Oxygen Species in Metabolic and Inflammatory Signaling. Circ Res. 2018; 122:877–902. 10.1161/CIRCRESAHA.117.311401 29700084 PMC5926825

[36] Lennicke C, Cochemé HM. Redox metabolism: ROS as specific molecular regulators of cell signaling and function. Mol Cell. 2021; 81:3691–707. 10.1016/j.molcel.2021.08.018 34547234

[37] Silva L, Vargas C, Prados ME, Del Pozo A, Villa M, Martínez M, Alvarez L, Muñoz E, Unciti-Broceta JD, Martínez-Orgado J. Neuroprotective Efficacy of Betulinic Acid Hydroxamate, a B55α/PP2A Activator, in Acute Hypoxia-Ischemia-Induced Brain Damage in Newborn Rats. Transl Stroke Res. 2023; 14:397–408. 10.1007/s12975-022-01017-4 35419730

[38] Thornton C, Baburamani AA, Kichev A, Hagberg H. Oxidative stress and endoplasmic reticulum (ER) stress in the development of neonatal hypoxic-ischaemic brain injury. Biochem Soc Trans. 2017; 45:1067–76. 10.1042/BST20170017 28939695 PMC5652227

[39] Fuhrmann DC, Brüne B. Mitochondrial composition and function under the control of hypoxia. Redox Biol. 2017; 12:208–15. 10.1016/j.redox.2017.02.012 28259101 PMC5333533

[40] Perier C, Tieu K, Guégan C, Caspersen C, Jackson-Lewis V, Carelli V, Martinuzzi A, Hirano M, Przedborski S, Vila M. Complex I deficiency primes Bax-dependent neuronal apoptosis through mitochondrial oxidative damage. Proc Natl Acad Sci USA. 2005; 102:19126–31. 10.1073/pnas.0508215102 16365298 PMC1323177

[41] Li L, Lin Z, Yuan J, Li P, Wang Q, Cho N, Wang Y, Lin Z. The neuroprotective mechanisms of naringenin: Inhibition of apoptosis through the PI3K/AKT pathway after hypoxic-ischemic brain damage. J Ethnopharmacol. 2024; 318:116941. 10.1016/j.jep.2023.116941 37480970

[42] Comità S, Femmino S, Thairi C, Alloatti G, Boengler K, Pagliaro P, Penna C. Regulation of STAT3 and its role in cardioprotection by conditioning: focus on non-genomic roles targeting mitochondrial function. Basic Res Cardiol. 2021; 116:56. 10.1007/s00395-021-00898-0 34642818 PMC8510947

[43] Butturini E, Carcereri de Prati A, Mariotto S. Redox Regulation of STAT1 and STAT3 Signaling. Int J Mol Sci. 2020; 21:7034. 10.3390/ijms21197034 32987855 PMC7582491

[44] Zhu T, Meng XB, Dong DX, Zhao LY, Qu MW, Sun GB, Sun XB. Xuesaitong injection (lyophilized) combined with aspirin and clopidogrel protect against focal cerebral ischemic/reperfusion injury in rats by suppressing oxidative stress and inflammation and regulating the NOX2/IL-6/STAT3 pathway. Ann Palliat Med. 2021; 10:1650–67. 10.21037/apm-20-1681 33222458

[45] Huo S, Shi W, Ma H, Yan D, Luo P, Guo J, Li C, Lin J, Zhang C, Li S, Lv J, Lin L. Alleviation of Inflammation and Oxidative Stress in Pressure Overload-Induced Cardiac Remodeling and Heart Failure via IL-6/STAT3 Inhibition by Raloxifene. Oxid Med Cell Longev. 2021; 2021:6699054. 10.1155/2021/6699054 33824698 PMC8007383

[46] Zhao J, Liu X, Chen Y, Zhang LS, Zhang YR, Ji DR, Liu SM, Jia MZ, Zhu YH, Qi YF, Lu FM, Yu YR. STAT3 Promotes Schistosome-Induced Liver Injury by Inflammation, Oxidative Stress, Proliferation, and Apoptosis Signal Pathway. Infect Immun. 2021; 89:e00309–20. 10.1128/IAI.00309-20 33257536 PMC8097265

[47] Mehla J, Singh I, Diwan D, Nelson JW, Lawrence M, Lee E, Bauer AQ, Holtzman DM, Zipfel GJ. STAT3 inhibitor mitigates cerebral amyloid angiopathy and parenchymal amyloid plaques while improving cognitive functions and brain networks. Acta Neuropathol Commun. 2021; 9:193. 10.1186/s40478-021-01293-5 34911575 PMC8672532

[48] Hristova M, Rocha-Ferreira E, Fontana X, Thei L, Buckle R, Christou M, Hompoonsup S, Gostelow N, Raivich G, Peebles D. Inhibition of Signal Transducer and Activator of Transcription 3 (STAT3) reduces neonatal hypoxic-ischaemic brain damage. J Neurochem. 2016; 136:981–94. 10.1111/jnc.13490 26669927 PMC4843952

[49] Xiong L, Zhou H, Zhao Q, Xue L, Al-Hawwas M, He J, Wu M, Zou Y, Yang M, Dai J, He M, Wang T. Overexpression of miR-124 Protects Against Neurological Dysfunction Induced by Neonatal Hypoxic-Ischemic Brain Injury. Cell Mol Neurobiol. 2020; 40:737–50. 10.1007/s10571-019-00769-2 31916069 PMC11448850

[50] Beurel E, Jope RS. Lipopolysaccharide-induced interleukin-6 production is controlled by glycogen synthase kinase-3 and STAT3 in the brain. J Neuroinflammation. 2009; 6:9. 10.1186/1742-2094-6-9 19284588 PMC2660311

[51] Kim H, Leng K, Park J, Sorets AG, Kim S, Shostak A, Embalabala RJ, Mlouk K, Katdare KA, Rose IVL, Sturgeon SM, Neal EH, Ao Y, et al. Reactive astrocytes transduce inflammation in a blood-brain barrier model through a TNF-STAT3 signaling axis and secretion of alpha 1-antichymotrypsin. Nat Commun. 2022; 13:6581. 10.1038/s41467-022-34412-4 36323693 PMC9630454

[52] Priego N, Zhu L, Monteiro C, Mulders M, Wasilewski D, Bindeman W, Doglio L, Martínez L, Martínez-Saez E, Ramón YC S, Megías D, Hernández-Encinas E, Blanco-Aparicio C, et al. STAT3 labels a subpopulation of reactive astrocytes required for brain metastasis. Nat Med. 2018; 24:1024–35. 10.1038/s41591-018-0044-4 29892069

[53] Reisinger SN, Sideromenos S, Horvath O, Derdak S, Cicvaric A, Monje FJ, Bilban M, Häring M, Glat M, Pollak DD. STAT3 in the dorsal raphe gates behavioural reactivity and regulates gene networks associated with psychopathology. Mol Psychiatry. 2021; 26:2886–99. 10.1038/s41380-020-00904-2 33046834 PMC8505245

[54] Viesselmann C, Ballweg J, Lumbard D, Dent EW. Nucleofection and primary culture of embryonic mouse hippocampal and cortical neurons. J Vis Exp. 2011; 47:2373. 10.3791/2373 21304471 PMC3182630

[55] Rice JE 3rd, Vannucci RC, Brierley JB. The influence of immaturity on hypoxic-ischemic brain damage in the rat. Ann Neurol. 1981; 9:131–41. 10.1002/ana.410090206 7235629

[56] Ke F, Wang H, Geng J, Jing X, Fang F, Fang C, Zhang BH. MiR-155 promotes inflammation and apoptosis via targeting SIRT1 in hypoxic-ischemic brain damage. Exp Neurol. 2023; 362:114317. 10.1016/j.expneurol.2023.114317 36608839

[57] Ma Q, Dasgupta C, Shen G, Li Y, Zhang L. MicroRNA-210 downregulates TET2 and contributes to inflammatory response in neonatal hypoxic-ischemic brain injury. J Neuroinflammation. 2021; 18:6. 10.1186/s12974-020-02068-w 33402183 PMC7786974

[58] Li Y, Zhang Y, Walayat A, Fu Y, Liu B, Zhang L, Xiao D. The Regulatory Role of H19/miR-181a/ATG5 Signaling in Perinatal Nicotine Exposure-Induced Development of Neonatal Brain Hypoxic-Ischemic Sensitive Phenotype. Int J Mol Sci. 2022; 23:6885. 10.3390/ijms23136885 35805891 PMC9266802

[59] Heyser CJ. Assessment of developmental milestones in rodents. Curr Protoc Neurosci. 2004; 8:8.18. 10.1002/0471142301.ns0818s25 18428605

[60] Castelhano-Carlos MJ, Sousa N, Ohl F, Baumans V. Identification methods in newborn C57BL/6 mice: a developmental and behavioural evaluation. Lab Anim. 2010; 44:88–103. 10.1258/la.2009.009044 19854756

